# A look at the normal development of Pointing and Reaching Gestures in 12-16-Month-Old Farsi-Speaking Children: A Longitudinal Study

**Published:** 2018

**Authors:** Zahra BABAEI, Talieh ZARIFIAN, Atieh ASHTARI, Enayatollah BAKHSHI, Mona EBRAHIMPOUR

**Affiliations:** 1 Department of Speech Therapy,University of Social Welfare and Rehabilitation Sciences, Tehran, Iran.; 2 Statistics Department, University of Social Welfare and Rehabilitation Sciences, Tehran, Iran

**Keywords:** Gesture, Pointing, Reaching, Development, Iranian children

## Abstract

**Objectives:**

Human beings can use gestures such as pointing and reaching to communicate with others before they have the ability to use verbal communication to produce speech. Given the importance of children's communication development and the key role of gestures development in communicating, the main purpose of this study was to analyze the normal development of pointing and reaching gestures and their relationship in 12-16-month-old children speaking Farsi.

**Materials & Methods:**

In this prospective, observational and longitudinal study the gestures of 11 monolingual Farsi-speaking children (7 boys and 4 girls, from Oct 2015 to Jan 2017 in the homes of participants across Tehran, Iran) were evaluated via non-randomized sampling method. Child-mother interactions were videotaped monthly in a semi-structured context to capture the emergence and consistent use of targeted gestures. Afterward, the data were coded and statistically analyzed for this purpose Repetitive measured; independent *t*-test and Pearson correlation were used.

**Results:**

The mean of the pointing gesture increased significantly from 12 to 16 months (*P*<0.05). However, this was not significant for the reaching gesture. Moreover, there was no relationship between pointing and reaching gestures.

**Conclusion:**

Pointing gestures increase with age from 12-16 months in Farsi-speaking children. However, reaching gestures stay the same between 12-16 months of age. The study provided rich details of common gestures that children use to signal their intentions before verbal communication.

## Introduction

Human beings are social creatures and have a strong tendency to communicate even in the first months of life. This communication can have different communicative functions such as behavior regulation, social interaction and joint attention (1-3). Infants use a variety of gestures to communicate with others. Gestures are movements of parts of the body, especially the hands or head to express a meaning or a need (2-5). Children develop intentional communication around 8 to 9 months and they use gestures to meet their needs (6, 7). Pointing is one of the main gestures which are extending the finger toward something (8). It enables the child to communicate with others before accessing the verbal skills (2, 3). The pointing gesture is a tool to regulate other's behavior results from more primitive gestures known as reaching gesture (9-12). Reaching gesture is extending a hand toward an object except grasping movements (8). “Most basically, infant's acts of pointing are underpinned by: i) motoric prerequisites for index finger extension toward external objects; ii) motivational prerequisites for communicating with others in various ways (e.g requesting things imperatively, or indicating them declaratively); and iii) social-cognitive prerequisites for following, directing, and sharing attention with others” (13). According to classic theories, infants' motivation to point for others begin with their attempt to obtain out-of-reach objects, on the other hand, human beings' fine motor skills develop with increasing the age, and one of the main skills is Index finger extension adapted for pointing gesture. In other hand, by increasing the age, infants imitate their caregivers' pointing gestures for communication and learn to point for achieving their intentions. Therefore, reaching gesture with the imperative function (e.g. Behavior regulation) converts to the pointing gesture with the same function by increasing the age, so decreasing of reaching and increasing of pointing gestures are expected (10, 12-16). Reaching gesture appears first in the development and is the most frequent gesture during the first year of life (17). According to most studies in various societies, pointing gesture increase in the second year of life, especially in 16-20 months of age (3, 8, 18-23).

Because of the time period importance in diagnosis and early intervention, the study of gesture, as one of basic indices of evaluation and early intervention of language disorders and communication problems can guide clinicians to diagnose developmental problems timely (24, 25).

“Using natural gestures in promoting communication and linguistic skills in children with prelinguistic communication disorders has become essential, but it requires deeper knowledge” (20). Although many studies have been done on the development of these gestures, there is contradictory evidence about their developmental stages and their frequency. For instance, the highest frequency of reaching gestures was reported in 9-15 months, while this increase has been reported in 8-12 months in another survey (20, 26). On one hand, this increase in reaching gestures happens in 9.5-12.5 months (18). On the other hand, reaching gestures in Chinese stabilize in 8-14 months and even reduce in some 12-21 month-old infants (19). There are different reports on the pointing developmental process such as reaching gesture. For example, the highest frequency of pointing gesture was reported after 11 months (27), while this increase has been reported in 16-20 months (22, 23). Pointing gesture frequency increase as children's spoken labeling behavior promote in 18-20 months. Some scientists contribute these differences in the social-pragmatic aspects of communication and the effect of culture on that (28, 29), these attitudes are correlated to system theories, according to these theories a child's development is shaped by the varied systems and human beings develop within a system of relationships that include family and society (30). Therefore, the norms in one society cannot be representative of the norms in another society, and the milestones of these prelinguistic behaviors must be determined in every. That is why gestures have been studied in 30 languages in the world in less than a century (3, 28, 29, 31-33).

Children usually start naming an item 2-3 months after pointing to it. In fact, pointing gesture is a prerequisite for the development of vocabulary and lexical enrichment (18, 34). Moreover, the use of combination of gesture and spoken word can predict two-word utterances in children (35).

The relationship between reaching gesture and the development of language skills, especially the number of receptive and expressive words in the first year of life has been proven (18). In spite of this fact, reaching gesture is negatively correlated with the use of gestures in children with the use of language in older children (20).

Using gesture in children with communication and language impairment is not only different from their normal peers, but also has distinct properties in different developmental disorders (5, 36-47).

Considering the relationship between language development and use of gestures (17), a better understanding of gestures' milestones, stages, and frequency appears to be essential. Consequently, clinicians will be able to diagnose and conduct early intervention more effectively. Although there are numerous studies on the development of gestures (3, 4, 28, 29, 32, 33, 42-47) all of them are conducted in other language and culture, so there is no evidence on this topic in Iranian children. 

Therefore, the main purpose of this study was to examine and describe the frequency, developmental process, and relationships of pointing and reaching gestures in 12-16-month-old toddlers in Iran.

## Materials & Methods

The study was conducted from Oct 2015 to Jan 2017 in the homes of participants across Tehran, Iran.

This was a prospective, observational and longitudinal study in which the gestures of 11 monolinguals (12-month) Farsi- speaking children (7 boys and 4 girls) were evaluated. Statistical power for this sample size was considered (as) 80%. All participants were medically normal, had normal development, their parents had at least 12 yr. of formal education, and they were monolingual and had a moderate socioeconomic status (48). Ages & Stages Questionnaire (ASQ) was administered to select typically-developed children. Children and their parents who met the inclusion criteria were recruited. 

Sampling was initially done through snowball method which is a non-probability sampling technique. Four subjects were recruited from health centers in moderate Socio-economic status areas of the town; four subjects were introduced by other researchers, and three families were referred by other participants in this study. 

 The data collection instruments included mother-child demographic inventory, ASQ, and handy cam.

Mother-child demographic inventory included general question (child's sex, birth order, medical history and mother education and age), the validity of the demographic questionnaire was evaluated through content validity using scientific resources and expert's opinion. The content validity of questionnaire was qualitatively determined. The questions were revised based on the 14 speech-language Pathologists and then were presented to the parents.

ASQ is the most widely used for developmental disorders determination given to mothers to be filled. ASQ (3^rd^ ed) includes 19 different questionnaires which evaluate communication, gross motor, fine motor, problem-solving and personal-social skills in 4 to 60-month-old children. Each questionnaire has 30 questions, and each domain has 6 questions. ASQ was validated and normalized on Iranian children living in Tehran (49). The Cronbach's alpha for the questionnaire is overall 0.79 and the inter-rater reliability was 0.93. The constant validity of the questionnaire was also confirmed by the factor analysis. The Persia version of ASQ can validly and reliably screen developmental disorders in Iranian children residing in Tehran (49).

The child-mother interactions were recorded using a digital video recorder (Sony.HDR-XR100, Model no:AC-L200C).

After explaining the process of the study and its objectives for the families, a written consent was signed by them, and then the examiner conducted the assessment at the child's home at their convenience. At the beginning of the first session, information about the child's interests and communication methods (behaviors) such as gestures were gathered from the family. Afterward, the mother was explained about how to play and interact with the child in order to evoke the maximum communication act in the child. The mother-child interaction was videotaped by the Sony handy cam (HDR-XR100). The interaction begins with 15-min play with the child's toys and then move to 45-min play with the designed toys (Appendix 1). This procedure continued on a monthly basis for 5 months. Videos of each session were recorded by the researcher through the anecdotal method, and then all the pointing and reaching gestures (during 1-h child-mother interaction) were coded (Appendix 2). 

The coded data were analyzed using SPSS ver. 22 (Chicago, IL, USA) and the descriptive statistics such as mean, and standard deviation. As the data were normal according to Shapiro-Wilk Test, analytic statistics such as repetitive measured (within-subject comparison), independent t-test was used.

In order to validate the coding system, 20% of the recorded samples were coded by two examiners educated earlier on coding and scoring the gestures and had experience in the development of gestures.

This research was confirmed by the Ethics Committee of the University of Social Welfare and Rehabilitation Sciences, Tehran, Iran. The frequency of pointing and reaching gestures is shown in Table 2.

## Results

The demographic information of the participants is presented in Table 1.

**Table 1:** Demographic information

Child	K.A	R.M	A.M	M.M	A.Sh	P.A	Z.T	Z.A	M.H	Q.H	M.R
Data											
Sex	Male	Male	Male	Male	Female	Male	Female	Female	Male	Female	Male
Age (day)	360	361	362	360	363	362	361	363	363	363	364
Birth order	1	3	1	2	1	1	1	3	3	1	2
Mother education	Master of science	Diploma	Bachelor	Bachelor	Bachelor	Bachelor	Master of science	Diploma	Diploma	Master of science	Diploma
Mother age	28	36	27	33	24	27	29	32	33	29	32

**Table 2:** Descriptive statistics of the pointing and reaching gestures

Age	12 months	13 months	14 months	15months	16 months
Gestures					
Pointing gesture frequency (standard deviation)	12.000 (9.979)	15.272 (14.519)	29.545 (28.380)	29.272 (22.249)	30.909 (21.505)
Reaching gesture frequency (standard deviation)	22.545 (4.131)	21.909 (8.431)	25.909 (10.940)	24.636 (8.969)	21.636 (9.718)

**Table 3:** Repetitive measurements (within-subject comparison) of the pointing and reaching gestures in 2 successive months

Gesture		Mean Square	F	Sig.
Pointing	12-13	117.818	1.059	0.328
	13-14	2240.818	9.478	0.012*
	14-15	0.818	0.003	0.956
	15-16	29.455	0.049	0.830
Reaching	12-13	4.455	0.066	0.802
	13-14	176.000	1.252	0.289
	14-15	17.818	0.166	0.693
	15-16	99.000	0.870	0.373

* (*P*<0.05) is significant

**Table 4:** Independent *t*-test of the pointing and reaching gestures in comparing two consecutive months

Gesture		Df	T	Sig
Pointing	13	17.725	0.616	0.546
	14	12.436	1.934	0.076
	15	13.868	2.349	0.034*
	16	14.116	2.645	0.019*
Reaching	13	14.541	-0.225	0.825
	14	12.796	0.954	0.358
	15	14.061	0.702	0.494
	16	13.501	-0.286	0.780

* (*P*<0.05) is significant

**Figure 1:**
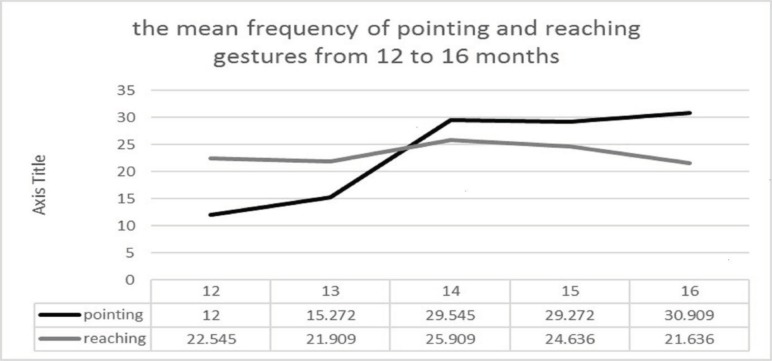
The mean frequency of pointing and reaching gestures from 12-16 months

Another purpose of this study was to determine the developmental process of pointing and reaching gestures. As illustrated in Table 2 and Figure 1, the frequency of pointing gesture incline monthly with age, except in 14-15 months in which no significant difference was observed (*P*>0.05). The findings from the repetitive measurements in 2 successive months revealed that there is no significant difference, except in 13-14 months in which significant difference was observed (*P*=0.012). However, if we were comparing two consecutive months, the difference in the mean frequency of the pointing gestures would be significant (12-15 months (*P*=0.034); 12-16 months (*P*=0.019)), as it was shown in Table 3 and 4.

According to the Table 2 and Figure 1, the frequency of reaching gesture followed the upward trend monthly, and the findings from the repetitive measurement in two successive months revealed that there was no meaningful difference between the frequency means of reaching gestures. Generally, the comparison of the month 12 with other months revealed that there was no significant difference in the reaching gestures. Although the chart 2 shows a subtle decline in the frequency of reaching gestures, this decline was not statistically significant.

The third purpose of this research was to study the relationship between pointing and reaching gestures. The results of Pearson correlation indicated that there was no meaningful relationship between these two gestures.

However, inter-examiner reliability was examined on the 20% of the samples by two examiners. The intra-class correlation coefficient (ICC) was 90% (0.9) in this evaluation.

**Appendix 1:** The designed toys in this study include:

The designed toys
Bubble blower, puppet, baby doll, balloon, hairbrush, cups, spoons, plates, pan, baby bottle, toothbrush, towel, book, blanket or facecloth for peek-a-boo.

**Appendix 2:** The operational definitions for pointing and reaching gestures

Pointing	Point with index finger Point with an object in hand Touchpoint, when index finger touches the referent Touchpoint with motion, when index finger touches the referent and moves but remains in contact with the referent. Touch and tap, when index finger touches the referent-lifts off the referent and touches referent again. Whole hand touch
Reaching	Reaching with one or both arms extended toward an object that is out of reach. Partial reach, when one or both arms are partially extended. Reach to take Reach with open hand or opening and closing hand

## Discussion

The main purpose of this study was to determine the frequency and the typical development of pointing and reaching gestures and their relationship in Farsi-speaking children aged 12-16 months. Pointing and reaching gestures at the child's first communication means prior to the verbal communication stage (6, 7), and can be used by pediatricians and clinicians to screen the developmental problems in children. This, as a developmental milestones scale, can also predict language and communication development (24). 

Based on our results, the mean of the pointing gesture increased significantly from 12 to 16 months (*P*<0.05). However, this was not significant for the reaching gesture. Moreover, there was no relationship between pointing and reaching gestures, considering the statistical power of 80%, this might be because of our small sample size.

Our results about pointing gesture consist with another study showing that pointing gesture increase during the second year of life (3, 5, 8, 18, 22, 28, 50). A possible explanation for this phenomenon is that the child's ability (skill) for the three-dimensional interaction (mother, child, object) increases with age and combines with developing the joint attention. Pointing gesture is one of the most common methods of communicating with others; it is expected to increase with age. Another explanation for this might be found in mother's behavior. Mothers as the first and most influential teachers, usually use a combination of pointing gesture and verbal production for naming the objects and events. Children, similarly, 12-month old infants start using a combination form of gesture and word based on their experiences from the environment. The pointing is the most frequent gesture. Children use gestures in combination with their word production, our findings on increasing use of pointing gesture from 12-16 months seems logical (8, 22, 51, 52).

Moreover, we found that although there is a subtle difference in pointing gesture between successive months, these changes are not statistically substantial. This is probably because of a short distance between evaluation sessions. A dramatic change in the pointing gestures during only one month of development is not expected. However, the mean frequency of the pointing gesture increased significantly from 13 to 14 months (*P*=0.012). This change might be because of mother's more inclination toward using pointing gestures during this age (7, 50).

In addition to the pointing gesture, reaching gesture was also examined in this study. The results of our study indicated that reaching gesture did not change from 12 to 16 months of age, which is consistent with a study on reaching gestures in 8-14-month-old children (19). The hypothesis of this study was that reaching gesture with the imperative function (e.g. Behavior regulation) converts to the pointing gesture with the same function with increasing the age and developing the fine motor movements. Therefore, reaching gestures were expected to decrease with age. However, this hypothesis was not supported by our results. The methodology used in this study might have led us to this result. 

Encouraging communication tool was available for the child in this study; that, the child received the object from the mother (communicative partner), and there was no need and no motivation for pointing gesture (10, 12-14). Because the communication context was such that the infants preferred to use easier and faster means of communication and attending to their goals, so they used reaching gestures for imperative functions instead of using pointing gestures. On the other hand, infants learn to point for out-of-reach objects, not for available objects. Our small sample size might be another reason for this observation. Pointing gesture's developmental process was independent of reaching gesture, they developed in parallel to each other. The communication context was the most important component in using communication means (type of gestures), such an account is taken as support for proposal that culture can also have an effect on choosing and designing communication contexts and communication tools and consequently on communication means infants use.

The present study was the first longitudinal in which the development of reaching and pointing gestures and their relationship was analyzed in typical Farsi-speaking toddlers. The small sample size, individual differences between participants and their communicative partners lead us to be cautious about generalizing the findings of this study to all Iranian population. 

**In conclusion,** studying and making conclusions from a much broader population than our sample and examining these gestures in different races are recommended. The relationship between gesture development and communication act be examined in children with communicative disorders. Furthermore, the role of communicative partners, especially mothers, in the development of these gestures should be studied.
